# Genetic Evidence for Protective Effects of Angiotensin-Converting Enzyme Against Alzheimer Disease But Not Other Neurodegenerative Diseases in European Populations

**DOI:** 10.1212/NXG.0000000000200014

**Published:** 2022-08-29

**Authors:** David K. Ryan, Ville Karhunen, Bowen Su, Matthew Traylor, Tom G. Richardson, Stephen Burgess, Ioanna Tzoulaki, Dipender Gill

**Affiliations:** From the Clinical Pharmacology Group (D.K.R., D.G.), Pharmacy and Medicines Directorate, St George's University Hospitals NHS Foundation Trust; Clinical Pharmacology and Therapeutics Section (D.K.R., D.G.), Institute of Medical and Biomedical Education and Institute for Infection and Immunity, St George's, University of London; Centre for Clinical Pharmacology and Therapeutics (D.K.R.), University College London; Department of Epidemiology and Biostatistics (V.K., B.S., I.T., D.G.), School of Public Health, Imperial College London, United Kingdom; Research Unit of Mathematical Sciences (V.K.), University of Oulu; Center for Life Course Health Research (V.K.), University of Oulu, Finland; Clinical Pharmacology (M.T.), William Harvey Research Institute, Queen Mary University of London; The Barts Heart Centre and NIHR Barts Biomedical Research Centre-Barts Health NHS Trust (M.T.), The William Harvey Research Institute, Queen Mary University London; Novo Nordisk Research Centre Oxford (M.T., T.G.R., D.G.), Old Road Campus, Oxford; Medical Research Council Integrative Epidemiology Unit (T.G.R.), University of Bristol; Medical Research Council Biostatistics Unit (S.B., D.G.), Cambridge Institute of Public Health; Cardiovascular Epidemiology Unit (S.B.), Department of Public Health and Primary Care, University of Cambridge, United Kingdom; and Department of Hygiene and Epidemiology (I.T.), University of Ioannina, Greece.

## Abstract

**Background and Objectives:**

Angiotensin-converting enzyme (ACE) inhibitors are a commonly prescribed class of medication used to treat heart failure, hypertension, and chronic kidney disease. However, previous observational studies have shown conflicting directions of associations between ACE inhibitors and risk of Alzheimer disease. Genetic evidence has supported a protective effect of cerebral ACE against Alzheimer disease (AD). However, it is unclear whether this effect is mediated through blood pressure and extends to other neurodegenerative diseases.

**Methods:**

We performed genetic colocalization investigating an effect of cortical *ACE* expression on AD risk in people of European ancestry. We further investigated whether any effect of *ACE* expression on AD risk is mediated through changes in blood pressure and whether effects extend to Parkinson disease, small-vessel disease, or cognitive function in a Mendelian randomization paradigm.

**Results:**

There was genetic evidence supporting a protective effect of cortical *ACE* expression on AD risk in people of European ancestry. Although higher cortical *ACE* expression was associated with higher blood pressure, there was no strong evidence to support that its association with AD was mediated through blood pressure nor that *ACE* expression affected risk of other neurodegenerative traits.

**Discussion:**

Genetic evidence supports protective effects of cerebral ACE expression on AD, but not other neurodegenerative outcomes in people of European ancestry. Further work is required to investigate whether therapeutic inhibition of ACE increases risk of Alzheimer disease.

Alzheimer disease (AD) is a leading cause of morbidity worldwide, and its prevalence is projected to increase in line with the aging global population.^1^ Several preclinical and observational studies have implicated the role of CNS angiotensin-converting enzyme (ACE) levels in the pathogenesis of AD. Cerebral ACE and downstream product angiotensin II are increased in patients with AD and promote neuroinflammatory cytokines, reduce acetylcholine release, and attenuate cerebral blood flow—all factors implicated in the development of AD.^2^ Animal models have shown that hypertensive rats treated with centrally acting ACE inhibitors (e.g., captopril and perindopril), but not hydralazine, have significantly lower age-related impairment in learning and memory, regardless of changes in blood pressure.^3^ Observational data also support the neuroprotective role of central-acting ACE inhibitors compared with predominantly peripherally acting ACE inhibitors.^2,4^

On the contrary, there is also some evidence that ACE may serve in preventing AD. For example, in vitro studies have supported that ACE degrades amyloid-β plaques, a pathologic hallmark of AD.^5^ Animal AD models with heterozygous deletion of the *ACE* gene demonstrated that a decrease in ACE levels promoted amyloid-β deposition and increased the number of apoptotic neurons.^4^ At present, there is, therefore, uncertainty surrounding the role of ACE inhibitors in the pathogenesis of AD.

Genetic data, in the form of genome-wide association studies (GWASs), can be leveraged in a paradigm known as Mendelian randomization (MR) to study the causal effect of an exposure on an outcome. MR uses genetic polymorphisms to proxy the exposure in question and has notable benefits, including reduction in bias from confounding and reverse causality. This occurs because of the random allocation of genetic variants and balancing of confounding factors at conception. It also enables us to study the effects of long-term changes in exposure on life-time risk of a disease, which is advantageous in the context of neurodegenerative traits, which oftentimes develop over many years. This form of study design makes several assumptions: The genetic proxy must be associated with the exposure; the genetic variant only affects the outcome through the exposure of interest with no horizontal pleiotropic effect, and the genetic variant is not associated with any known confounder affecting the exposure and the outcome.

Recent GWAS have identified the *ACE* gene as a locus of interest in the development of AD.^6^ Bivariate GWAS and colocalization studies suggest that the *ACE* gene may mediate an association between blood pressure traits and AD risk, with the allele associated with lower SBP also associated with higher AD risk. Tissue-specific expression has demonstrated that higher cerebellar *ACE* expression has an association with AD risk.^7^ By extension, this implicates a possible detrimental effect of centrally acting pharmacologic ACE inhibition on AD risk. This is of direct clinical relevance because ACE inhibitors are one of the most commonly prescribed antihypertensive agents and are oftentimes commenced as a first-line medication in younger patients. Therefore, it is imperative to understand any potential long-term effect of ACE modulation on risk of later life neurodegenerative diseases.

To further explore the relationship between ACE and neurodegenerative diseases, we advance previous work by performing 3-way colocalization analyses for *ACE* gene expression in the cortex, systolic blood pressure (SBP), and AD risk. This approach enables us to identify a genetic proxy for the effect of ACE inhibitors that cross the blood-brain barrier and explore its association with risk of AD and other neurodegenerative diseases in a MR paradigm. Finally, we assess whether any association could be mediated by effects of SBP on AD risk. In this way, we elucidate the complex interplay between ACE and AD and assess the potential effect of ACE inhibition on neurodegenerative disease risk more widely.

## Methods

### Data Sources

For both colocalization and MR analyses, data were obtained from publicly available summary statistics of genome-wide association studies (GWASs) outlined in Table 1. We selected Parkinson disease, cognitive performance, lacunar stroke, and MRI quantified white matter hyperintensity as outcomes to represent common neurodegenerative conditions. Lacunar stroke and MRI-quantified white matter hyperintensity, both which are well-recognized features of vascular dementia, were used as surrogate outcomes in the absence of available GWAS for vascular dementia. Finally, cognitive function was selected to proxy the effects of cortical ACE expression on disease-agnostic cognitive function. Cognition outcome data were obtained from a meta-analysis of the Cognitive Genomic consortium GWAS and UK Biobank. This study defined cognition using a general cognitive function “g,” calculated based on the first unrotated component extracted from a principal component analysis of individual test scores across a range of neuropsychological tests.^8^ The largest publicly available GWAS for each trait at the time of the study was selected for inclusion.

**Table 1:**
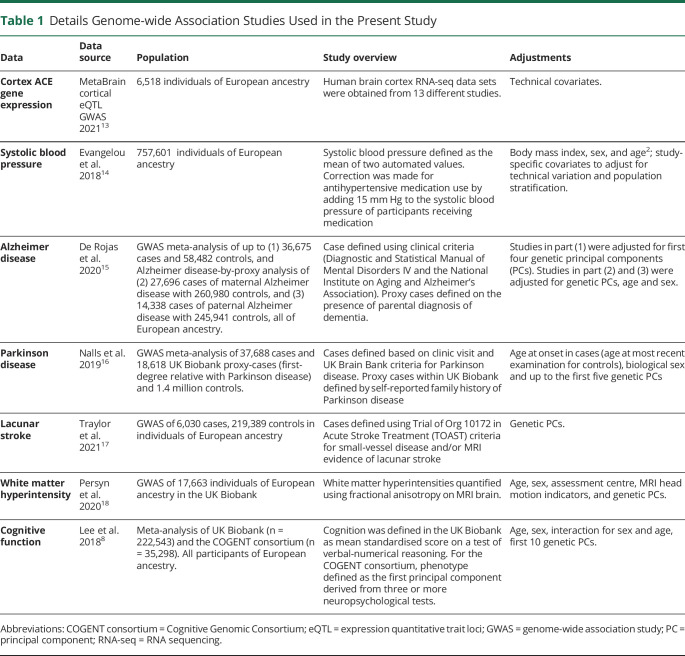
Details Genome-wide Association Studies Used in the Present Study

### Statistical Analysis

We conducted colocalization analysis of genetic associations for *ACE* gene expression in brain cortex tissue within±20 kb of the *ACE* gene (hg19 co-ordinates: chr17:61,554,422-61,599,205) and AD liability. In colocalization studies, we assess for the probability of a common shared variant between traits, which implies a likely shared causal mechanism. For pairwise colocalization between traits, we used coloc,^9^ a Bayesian method to calculate posterior probabilities (PPs) for the competing models:

1. Model 0: The genomic region does not contain a variant influencing the exposure or outcome.
2. Model 1: The genomic region contains a variant influencing only the exposure.
3. Model 2: The genomic region contains a variant influencing only the outcome.
4. Model 3: The genomic region contains two separate variants, one influencing the exposure and the other influencing the outcome.
5. Model 4: The genomic region contains a variant influencing both exposure and outcome.

The prior probability of any random variant being associated with both traits was set at 1 × 10^−5^. We defined colocalization between the exposure and outcome if the PP is greater than 0.8 for model 4.

We also assessed for 3-way colocalization with SBP using the HyPrColoc method^10^ (hypothesis prioritization for multitrait colocalization), to investigate whereby AD risk, cortical *ACE* expression, and blood pressure share a common causal variant. HyPrColoc estimates the posterior probability of full colocalization, that is, the probability of all traits sharing the same causal variant.^10^ The prior probability parameters in HyPrColoc were also set at their default values, i.e., the prior probability for a variant influencing one trait = 1 × 10^−4^ and the conditional probability of a variant influencing another trait = 0.02.^10^

The single-nucleotide polymorphism (SNP) with the greatest PP from colocalization analysis of cortical *ACE* expression and AD risk represents the genetic proxy most likely to simulate the effect of cerebral cortex ACE modulation on AD risk. We then use the MR paradigm to explore whether this variant is associated with other neurodegenerative traits.

MR uses genetic polymorphisms as instrumental variables to investigate the effects of an exposure on an outcome.^11^ For consistent causal estimates, the genetic proxy must (1) be associated with the exposure, (2) only affect the outcome through the exposure of interest, and (3) not associate with any confounder of the exposure and outcome.^11^

Finally, 2-sample MR was performed to investigate whether changes in blood pressure are responsible for mediating risk of AD and other neurodegenerative traits. Instruments to proxy systolic blood pressure (SBP) were selected as uncorrelated (r2 < 0.0001) variants that significantly associated with SBP (p < 5 × 10^−8^, selected instrumental variables detailed in eTable 1, links.lww.com/NXG/A540). Variants within the *ACE* gene were not removed for this because the aim of the analysis was to look at generic reduction in SBP, regardless of the gene of interest. Odds ratios were derived using inverse-variance weighted pooling of individual SNP Wald ratios, which corresponds to a weighted regression with the precisions of the variant-outcome associations acting as weights and the intercept fixed to zero. Sensitivity analysis was conducted to assess for potential pleiotropic effects using alternative pooling techniques (simple median, weighted median, and MR-Egger). Median estimates are robust even in situations where up to 50% of the weights contributing to the analysis are from invalid instruments.^12^ MR-Egger is a pooling technique where the intercept in the weighted regression is not fixed to zero. The intercept term is used to indicate the average pleiotropic effects of the variants used.^12^ A nonsignificant intercept term suggests no evidence for unbalanced pleiotropic effects. This method is robust even when all instrumental variables are invalid, as long as the Instrument Strength Independent of Direct Effect assumption holds, and any pleiotropic effect of the variants on the outcome are independent of the strength of their association with the exposure.^12^ All data analyses were performed using R statistical software, with “coloc” package version 4.1.0, “hyprcoloc” package version 1.0, and “TwoSampleMR” package version 4.26.

### Standard Protocol Approvals, Registrations, and Patient Consents

The sources of data used for this study are cited. All these studies obtained relevant participant consent and ethical approval. No formal ethical approval was required for use of publicly available genome-wide association data.

### Data Availability

All data used in this study are publicly available. The statistical code used in this work is available from the corresponding author upon reasonable request.

## Results

### Genetic Colocalization

**Figure 1:**
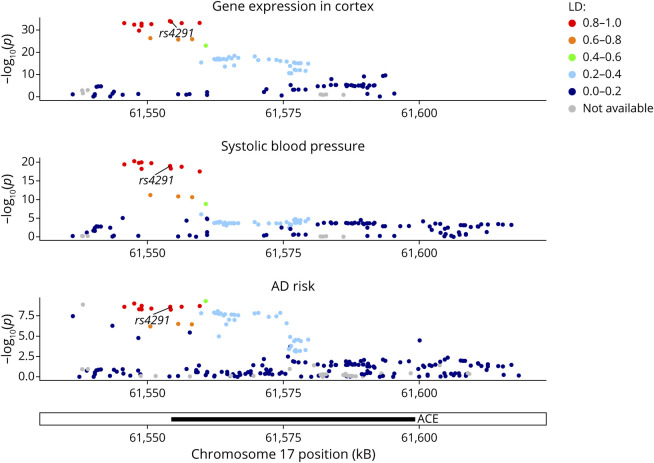
Colocalization Plots Depicting the Association of SNPs With Cortical *ACE* Gene Expression, Systolic Blood Pressure, and AD Risk The x-axis shows position within the genome (build Hg19) and y-axis denotes the −log_10_(*p*-value) for the association. Color denotes the LD between different variants (see legend). The results support that rs4291 is the most likely candidate SNP underlying all these traits, therefore, representing a common causal SNP for these traits. AD = Alzheimer disease; LD = linkage disequilibrium; SNP = single-nucleotide polymorphism.

Colocalization analysis provided evidence for a shared causal variant for each pairwise combination (PP for colocalization of cortical *ACE* gene expression and AD liability = 0.98; PP for colocalization of cortical *ACE* expression and SBP = 0.97; PP for SBP and AD liability = 0.98). In 3-way colocalization by HyPrColoc (Figure 1), the estimated PP of full colocalization = 0.83. The variant rs4291 was the most likely shared causal variant for all traits. Similar results were obtained when only considering AD cases and not also AD-by-proxy (eResults, links.lww.com/NXG/A540).

### Mendelian Randomization

**Figure 2:**
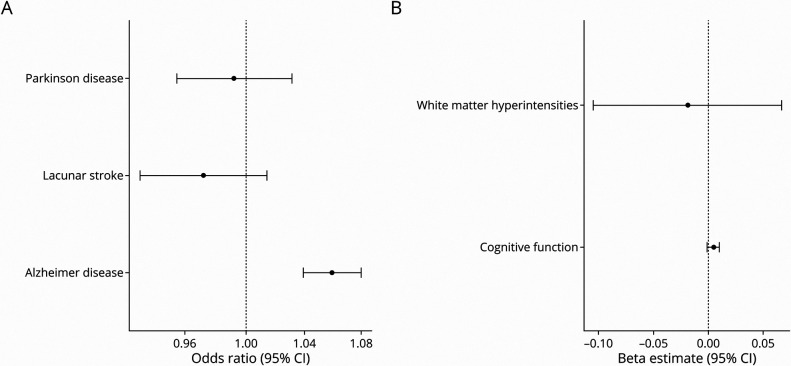
Associations Between Genetically Proxied ACE Inhibition and Neurodegenerative Traits Forest plot showing associations between genetically proxied ACE inhibition (rs4291 effect allele A) with (A) disease outcomes and (B) continuous traits. In graph A, the results reported as odds ratio per effect allele with 95% CI. The x-axis is presented on the log10 scale. For graph B, the results reported as beta estimate per effect allele with 95% CI. ACE = angiotensin-converting enzyme.

The A allele of rs4291 that associated with lower cortical *ACE* expression was negatively associated with SBP (effect estimate per increase −0.28 mm Hg, 95% CI −0.35 to −0.22). This same variant was positively associated with AD risk (odds ratio [OR] 1.06, 95% CI 1.04–1.08). However, strong associations were not identified for the other neurodegenerative traits (OR for lacunar stroke 0.97 [95% CI 0.93–1.01]; OR for Parkinson disease 0.99 [95% CI 0.96–1.03]; beta estimate for cognitive function 0.01 [95% CI −0.001 to 0.01]; beta estimate for white matter hyperintensity −0.02 [95% CI −0.11 to 0.07]; eTable 2, links.lww.com/NXG/A540, and Figure 2). Genetically predicted SBP was not associated with AD risk (OR 1.01, 95% CI 1.00–1.01 per mm Hg increase in SBP) using a genome-wide instrument, suggesting that although SBP colocalizes with cortical *ACE* expression at the *ACE* gene, reduction in SBP more generally is not directly associated with AD risk. These findings were consistent in sensitivity analysis (eTable 3).

## Discussion

This study leveraged genetic data to identify support for ACE in prevention of AD, with no strong evidence identified supporting effects of ACE on other neurodegenerative traits. Although increased cortical *ACE* expression is associated with lower AD risk, there was no MR evidence supporting that genetically predicted SBP affects risk of AD.

From a mechanistic perspective, ACE has been shown to breakdown neurotoxic amyloid-beta isoform (Aβ42) to a less toxic form (Aβ40). Administration of a clinical dose of ACE inhibitor to human amyloid precursor protein transgenic mice was associated with increased brain amyloid deposition.^4^ In humans, patients with AD have lower Aβ42-to-Aβ40–converting activity compared with sera from age-matched healthy individuals.^4^ Our current findings support that ACE protects against AD, although further work is required to investigate whether this is attributable to reduced amyloid aggregation or other unrelated mechanisms.

An observational study among 406 participants with mild-to-moderate AD demonstrated a reduction in cognitive decline for people receiving a centrally acting ACE inhibitor (perindopril) compared with peripherally acting ACE inhibitor.^19^ Other studies have shown increased risk of incident dementia and disability associated with peripherally acting ACE inhibitors compared with other antihypertensive medication.^20^ Conflicting findings between genetic and observational studies could be explained by MR being less liable to environmental confounding and reverse causality^11^ because of the random allocation of genetic variants at conception.

Our current work is consistent with other genetic studies supporting a role of ACE in preventing AD and has several additional strengths. First, obtaining association estimates from the MetaBrain consortium (n = 6,601 participants),^13^ we investigate cortical *ACE* expression and AD risk. This data set is significantly larger than the GTEx resource (n = 205) that has been used in previous work.^7^ Second, we investigate whether SBP mediates the relationship between ACE and AD risk and do not find evidence that supports this. Finally, we explore the associations of genetically proxied cortical *ACE* expression with other traits and do not find evidence to support that this association applies across other neurodegenerative traits.

This work also has several limitations. Clinical diagnosis of AD is challenging because there is significant overlap in symptoms with other forms of dementia, limiting the specificity of case definitions in GWAS. To explore for this, we also assessed several other neurocognitive traits. Given the absence of strong evidence of ACE effects for these outcomes, it seems likely that our findings are specific for AD risk, rather than a generic effect on dementia or cognition. As with all studies leveraging genetic data, there remains the possibility of biological pleiotropy introducing confounding. It is also not possible to extrapolate the magnitude of clinical effect or required drug exposure for ACE inhibitors to represent a real-world risk for AD. Factors such as the ability of an ACE inhibitor to cross the blood-brain barrier may also shape AD risk and should be further studied. Furthermore, our work was based on data obtained from individuals of European genetic ancestry, and it is unclear whether these findings extend to other ethnic groups. A strength of the current study is that we assess for association between cortical ACE expression and various neurodegenerative traits. The outcome GWAS data are obtained from relatively homogenous populations, with most studies using UK Biobank data. The consideration of participants of European genetic ancestry avoids bias related to combining results from different ancestry groups.

In summary, although ACE inhibitors have numerous indications and are the cornerstone of hypertension, chronic kidney disease and heart failure management, this study finds evidence for a beneficial effect of cerebral cortex ACE in preventing AD. It would be premature to alter current clinical practice based on this evidence, and rather these findings should encourage further research into the effect of ACE inhibitors on AD risk.

### Study Funding

S. Burgess is supported by a Sir Henry Dale Fellowship jointly funded by the Wellcome Trust and the Royal Society (204623/Z/16/Z). This research was funded by United Kingdom Research and Innovation Medical Research Council (MC_UU_00002/7) and was supported by the National Institute for Health Research Cambridge Biomedical Research Centre (BRC-1215-20014). The views expressed are those of the authors and not necessarily those of the National Institute for Health Research or the Department of Health and Social Care. For the purpose of open access, the author has applied a Creative Commons Attribution (CC BY) license to any Author Accepted Manuscript version arising from this submission. funding

### Disclosure

T. G. Richardson and D. Gill are employed part-time by Novo Nordisk. The remaining authors have no conflicts of interest. Full disclosure form information provided by the authors is available with the full text of this article at Neurology.org/NG. disclosure

## Glossary

ACE: angiotensin-converting enzyme
AD: Alzheimers disease
GWASs: genome-wide association studies
MR: Mendelian randomization
OR: odds ratio
SNP: single-nucleotide polymorphism

## References

[1] Weller J, Budson A. Current understanding of Alzheimer's disease diagnosis and treatment. *F1000Res*. 2018;7(F1000 Faculty Rev):1161. 10.12688/f1000research.14506.1 PMC6073093 30135715

[2] Quitterer U, AbdAlla S. Improvements of symptoms of Alzheimer‘s disease by inhibition of the angiotensin system. Pharmacol Res. 2020;154:104230. 30991105 10.1016/j.phrs.2019.04.014

[3] Wyss JM, Kadish I, Van Groen T. Age-related decline in spatial learning and memory: attenuation by captopril. Clin Exp Hypertens. 2003;25(7):455-474. 14596369 10.1081/ceh-120024988

[4] Liu S, Ando F, Fujita Y, et al. A clinical dose of angiotensin-converting enzyme (ACE) inhibitor and heterozygous ACE deletion exacerbate Alzheimer's disease pathology in mice. J Biol Chem. 2019;294(25):9760-9770. 31072831 10.1074/jbc.RA118.006420 PMC6597817

[5] Hemming ML, Selkoe DJ. Amyloid β-protein is degraded by cellular angiotensin-converting enzyme (ACE) and elevated by an ACE inhibitor. J Biol Chem. 2005;280(45):37644-37650. 16154999 10.1074/jbc.M508460200 PMC2409196

[6] Schwartzentruber J, Cooper S, Liu JZ, et al. Genome-wide meta-analysis, fine-mapping and integrative prioritization implicate new Alzheimer's disease risk genes. Nat Genet. 2021;53(3):392-402. 33589840 10.1038/s41588-020-00776-w PMC7610386

[7] Bone WP, Siewert KM, Jha A, , et al; VA Million Veteran Program. Multi-trait association studies discover pleiotropic loci between Alzheimer's disease and cardiometabolic traits. Alzheimers Res Ther. 2021;13(1):34. 33541420 10.1186/s13195-021-00773-z PMC7860582

[8] Lee JJ, Wedow R, Okbay A, et al. Gene discovery and polygenic prediction from a genome-wide association study of educational attainment in 1.1 million individuals. Nat Genet. 2018;50(8):1112-1121. 30038396 10.1038/s41588-018-0147-3 PMC6393768

[9] Giambartolomei C, Vukcevic D, Schadt EE, et al. Bayesian test for colocalisation between pairs of genetic association studies using summary statistics. Plos Genet. 2014;10(5):e1004383. 24830394 10.1371/journal.pgen.1004383 PMC4022491

[10] Foley CN, Staley JR, Breen PG, et al. A fast and efficient colocalization algorithm for identifying shared genetic risk factors across multiple traits. Nat Commun. 2021;12(1):764. 33536417 10.1038/s41467-020-20885-8 PMC7858636

[11] Burgess S, Butterworth A, Malarstig A, Thompson SG. Use of Mendelian randomisation to assess potential benefit of clinical intervention. BMJ. 2012;345:e7325. 23131671 10.1136/bmj.e7325

[12] Burgess S, Thompson SG. Interpreting findings from Mendelian randomization using the MR-Egger method. Eur J Epidemiol. 2017;32(5):377-389. 28527048 10.1007/s10654-017-0255-x PMC5506233

[13] de Klein N, Tsai EA, Vochteloo M, et al. Brain expression quantitative trait locus and network analysis reveals downstream effects and putative drivers for brain-related diseases. bioRxiv. 2021. 10.1038/s41588-023-01300-6 PMC10011140 36823318

[14] Evangelou E, Warren HR, Mosen-Ansorena D, et al. Publisher Correction: genetic analysis of over 1 million people identifies 535 new loci associated with blood pressure traits. Nat Genet. 2018;50(10):1755. 30429575 10.1038/s41588-018-0297-3

[15] de Rojas I, Moreno-Grau S, Tesi N, et al. Common variants in Alzheimer's disease and risk stratification by polygenic risk scores. Nat Commun. 2021;12(1):3417-3516. 34099642 10.1038/s41467-021-22491-8 PMC8184987

[16] Nalls MA, Blauwendraat C, Vallerga CL, et al; System Genomics of Parkinson's Disease Consortium. , International Parkinson's Disease Genomics Consortium. Identification of novel risk loci, causal insights, and heritable risk for Parkinson's disease: a meta-analysis of genome-wide association studies. Lancet Neurol. 2019;18(12):1091-1102. 31701892 10.1016/S1474-4422(19)30320-5 PMC8422160

[17] Traylor M, Persyn E, Tomppo L, et al; Helsinki Stroke, Study Dutch Parelsnoer Institute-Cerebrovascular Accident CVA Study Group, National Institute of Neurological Disorders and Stroke NINDS Stroke Genetics Network, UK DNA Lacunar Stroke Study Investigators, International Stroke Genetics Consortium. Genetic basis of lacunar stroke: a pooled analysis of individual patient data and genome-wide association studies. Lancet Neurol. 2021;20(5):351-361. 33773637 10.1016/S1474-4422(21)00031-4 PMC8062914

[18] Persyn E, Hanscombe KB, Howson JMM, Lewis CM, Traylor M, Markus HS. Genome-wide association study of MRI markers of cerebral small vessel disease in 42, 310 participants. Nat Commun. 2020;11(1):2175. 32358547 10.1038/s41467-020-15932-3 PMC7195435

[19] O'Caoimh R, Healy L, Gao Y, et al. Effects of centrally acting angiotensin converting enzyme inhibitors on functional decline in patients with Alzheimer's disease. J Alzheimer’s Dis. 2014;40(3):595-603. 24496072 10.3233/JAD-131694

[20] Sink KM, Leng X, Williamson J, et al. Angiotensin-converting enzyme inhibitors and cognitive decline in older adults with hypertension: results from the cardiovascular health study. Arch Intern Med. 2009;169(13):1195-1202. 19597068 10.1001/archinternmed.2009.175 PMC2881686

